# New Insights into Chronological Mobility of Retrotransposons In Vivo

**DOI:** 10.1155/2019/2818415

**Published:** 2019-06-26

**Authors:** Amr. R. Ghanam, Jun Cao, Xuan Ouyang, Xiaoyuan Song

**Affiliations:** Hefei National Laboratory for Physical Sciences at the Microscale, CAS Key Laboratory of Brain Function and Disease, Neurodegenerative Disorder Research Center, School of Life Sciences, Division of Life Sciences and Medicine, University of Science and Technology of China, Hefei, Anhui 230026, China

## Abstract

Tissue aging is the gradual decline of physiological homeostasis accompanied with accumulation of senescent cells, decreased clearance of unwanted biological compounds, and depletion of stem cells. Senescent cells were cell cycle arrested in response to various stimuli and identified using distinct phenotypes and changes in gene expression. Senescent cells that accumulate with aging can compromise normal tissue function and inhibit or stop repair and regeneration. Selective removal of senescent cells can slow the aging process and inhibits age-associated diseases leading to extended lifespans in mice and thus provides a possibility for developing antiaging therapy. To monitor the appearance of senescent cells *in vivo* and target them, a clearer understanding of senescent cell expression markers is needed. We investigated the age-associated expression of three molecular hallmarks of aging: SA-*β*-gal, P16^INK4a^, and retrotransposable elements (RTEs), in different mouse tissues during chronological aging. Our data showed that the expression of these markers is variable with aging in the different tissues. P16^INK4a^ showed consistent increases with age in most tissues, while expression of RTEs was variable among different tissues examined. These data suggest that biological changes occurring with physiological aging may be useful in choosing the appropriate timing of therapeutic interventions to slow the aging process or keep more susceptible organs healthier in the aging process.

## 1. Introduction

Aging is a time-dependent decline of normal physiological processes. It is a phenomenon shared by almost all living organisms and results from the consequences of accumulations of or decreased clearance of biological compounds in senescent cells. Aging at the organism level increases an individual's susceptibility to disease, including cancers, metabolic disorders, diabetes, cardiovascular diseases, and neurodegenerative diseases [1–5]. On the other hand, replicative senescence, described by Hayflick as a permanent state of cell cycle arrest [6], is a process by which cells lose their proliferative potential and can serve as an endogenous anticancer strategy, resulting in irreversible growth arrest in response to potentially oncogenic stimuli [7]. Thus, senescence is a double-edged sword that has both beneficial and adverse effects.

Different research groups have identified several aging markers (reviewed in detail in [8]), including increased *β*-galactosidase (*β*-gal) reflecting the accumulation of lysosomes with aging [9]. Traditionally, tissue aging has been assessed by measuring the fraction of *β*-gal-positive cells [10].

P16^INK4a^ is one of the cyclin-dependent kinase inhibitors that drive the aging process, and it is considered another marker of aging. P16^INK4a^ activates the tumor suppressor, retinoblastoma (Rb), which enforces cell cycle arrest and induces senescence. Aged cells that do not express P16^INK4a^ may resume growth after inactivation of the tumor suppressor, P53 [11]. Accumulation of P16^INK4a^-positive senescent cells can induce tissue degeneration and cataracts in mice, while clearance of P16^INK4a^ senescent cells delayed aging and increased the average lifespan of mice [12, 13].

Transposable elements (TEs) are highly expressed in eukaryotic cells, representing nearly half of eukaryotic genomes, and persist through independent replication of their sequences. They move in the genome via RNA intermediates (retrotransposons) or direct cutting and pasting of their DNA sequences (DNA transposons), through the action of transposase, which is encoded in the sequence. Retrotransposons can be subdivided into two subgroups: those with long terminal repeats (LTRs) and those without LTRs (non-LTRs). LTR retrotransposons are widely found in eukaryotes and make up nearly 8% of the human genome [14]. The most abundant TEs in mammals are non-LTR retrotransposons, including long and short interspersed nuclear elements (LINEs and SINEs, respectively), both of which are widespread in eukaryotic genomes. LINE-1 (Long Interspersed Element 1) and Alu elements are two non-LTR retrotransposons that account for approximately one-quarter of the human genome. Retrotransposons can mobilize themselves through an element-derived RNA intermediate, which is converted into complementary DNA (cDNA) by reverse transcription. This is followed by integrating into a new genomic locus that generates a second copy of the TE, which is referred to as a copy-and-paste mechanism.

Organisms counteract transposition of retrotransposable elements (RTEs) by preventing their transcription through heterochromatinization of the genomic regions where they are inserted, thus repressing their activity [15], or by posttranscriptional actions of the Piwi-piRNA system that effectively silences TEs in germ cells and tumor cells. The piRNA pathway also can repress TE activity at the transcriptional level through RNA-mediated chromatin modification, which is referred to as RNA-mediated epigenetic silencing [16].

Based on studies from several model organisms, TEs can be highly active in the genome, raising the possibility that these mobile elements promote genomic instability and contribute to loss of cellular function with age. Furthermore, epigenetic studies of diverse aging models have revealed that TEs are associated with extensive relaxation of heterochromatin, decreased core histone content, altered posttranslational modifications, and genomic insatiability [17]. These observations led to a theory of aging through transposition [18]. TE misregulation also has been associated with specific diseases and disorders, such as cancers, aging [19], neurological disorders [20], and autoimmunity [21].

The extent of senescent cell accumulation in different organs of mammals in the aging process and the functional outcome of such accumulation are not very clear. In the current study, we examined three aging hallmarks, *β*-gal, P16^INK4a^, and RTEs, to verify the sequential order of aging in different mouse tissues throughout the aging process. Upon identification of age-susceptible organs, further studies will be able to direct to understand the underlying molecular mechanism(s) and potentially lead to the development of preventive therapies that target tissue-specific senescent cells.

## 2. Materials and Methods

### 2.1. Experimental Animals

The present work was carried out using healthy male BALB/c mice purchased from Vital River Laboratories (Beijing, China). We used young (1 mo), middle-aged (12 mo), and old mice (24 mo) that were maintained in specific-pathogen-free and humidity- and temperature-controlled microisolator cages with a 12-h light/dark cycle. Food and water were given *ad libitum*. The mice were anesthetized using chloral hydrate 8%, followed by cardiac infusion of phosphate-buffered saline (PBS), then different organs were dissected in cold PBS.

### 2.2. Ethics Committee Statement

All animal manipulations were conducted in strict accordance with the guidelines and regulations set forth by the University of Science and Technology of China (USTC) Animal Resources Center and University Animal Care and Use Committee. The protocol was approved by the Committee on the Ethics of Animal Experiments of the USTC (Permit Number: PXHG-SXY201510183) for mouse experiments.

### 2.3. MEF Cell Isolation

Following the methods of Ahmed et al. [22], uteri from pregnant female mice (Balb/C) at 14 days post coitum were dissected and washed with PBS and the embryos were collected into sterile Petri dishes with PBS. The fetal liver, heart, and head were removed, minced, and incubated at 37°C for 15 min in 0.25% trypsin EDTA (Gibco®, Grand Island, NY, USA) with gentle shaking as previously described [23]. Trypsin was neutralized with an equal amount of Dulbecco's modified Eagle's medium (DMEM), and the cells were collected by centrifugation (1000 rpm for 7 min). The isolated cells were resuspended and cultured on growth medium containing high glucose DMEM (11995-065, Gibco®) mixed with 10% fetal bovine serum (FBS, HyClone), 4 mM L-glutamine (25030-081, Gibco®), and 1 : 100 penicillin–streptomycin and incubated at 37°C with 5% CO_2_. We examined the cells daily using an inverted microscope (Olympus IX73, Japan).

### 2.4. *β*-Gal Assay

Tissue staining was performed as described previously [24]. Briefly, mouse tissues were rapidly dissected, immersed in OCT, then flash frozen using liquid nitrogen and sectioned at 5 *μ*M thickness using a cryostat (Leica CM1950). Sections were mounted on glass microscope slides, dried, rinsed with PBS, then fixed with 1% formaldehyde in PBS for 1 min at room temperature, followed by three PBS washes. The sections were stained overnight in *β*-gal solution (Beyotime C0602) at 37°C. Blue-stained cells were examined using an inverted microscope (Olympus IX73, Japan). Images were acquired using cellSens standard imaging software, followed by deconvolution and maximum protection under the default settings provided by the program software.

### 2.5. Western Blot Analysis

All steps were done according to the manufacturer's protocol, using a Bio-Rad system (Bio-Rad, USA). Briefly, each organ was cut into small pieces using scissors and homogenized in RIPA lysis buffer (150 mM NaCl, 1.0% NP40, 0.5% sodium deoxycholate, 0.1% SDS, 50 mM Tris-HCl, and pH 8.0 with protease inhibitors). The homogenized samples were slowly rotated at 4°C for 2 h followed by sonification for 5 min, then centrifuged at 12,000 g at 4°C for 10 min. The supernatant was collected, and the protein concentration was determined using a BCA protein assay kit (Beyotime, China). Protein samples were heated for 10 min at 95°C, then separated using 10% sodium dodecyl sulfate polyacrylamide gel electrophoresis (SDS–PAGE) (20 *μ*g protein/lane). Afterward, the samples were blotted onto polyvinylidene fluoride (PVDF) membranes (Solarbio, China) using a Trans-Blot SD system (Bio-Rad). The membranes with target proteins were blocked with BSA blocking buffer (CW2143S, CWBIO, China) for 2 hrs at room temperature, then incubated overnight with the primary antibody P16^INK4a^ (Proteintech, #10883-1-AP) and beta tubulin antibody (Proteintech, #66240-1-Ig) (1 : 2000) at 4°C. After three washes, the appropriate secondary antibody HRP labeled goat anti-mouse IgG or anti-rabbit IgG (Proteintech, #SA00001-1 and #SA00002-1) at a dilution of 1 : 5000. The membranes were incubated at room temperature for 2 hrs then washed using TBST (CW0043S, CWBIO, China). Bands were visualized using the enhanced chemiluminescence method (ECL) western blot kits (CW0048 M, CWBIO, China) and the FluorChem Q system (ProteinSimple, USA). Protein band intensity was quantified using the ImageJ program (National Institutes of Health, Bethesda, MD, USA).

### 2.6. Real-Time PCR Assessment of RTEs

Total RNA was extracted from the different mouse organs using TRIzol Reagent (Ambion, Cat. #: 15596) following the manufacturers' instructions. RNase-Free DNase (Promega, Cat. #: M6101) was used to eliminate DNA contamination. Reverse transcription was performed using the reverse transcription system (Promega, Cat. #: A5001). RT-qPCR was performed using 10 *μ*l aliquots and AceQ qPCR SYBR Green Master Mix (Vazyme # Q111-03) on a Bio-Rad detection instrument (CFX Connect), according to the manufacturer's specifications. Primers used for detection of different kinds of RTEs were previously described [25]. Real-time PCR primers for P16^INK4a^ and Mki67 were as follows: P16^INK4a^-F, 5′CGCAGGTTCTTGGTCACTGT3′; P16^INK4a^-R, 5′TGTTCACGAAAGCCAGAGCG3′; Mki67-F, 5′ATCATTGACCGCTCCTTTAGGT3′; Mki67-R, 5′GCTCGCCTTGATGGTTCCT3′; GAPDH-F, 5′ACATCATCCCTGCATCCACTG3′, GAPDH-R, 5′CCTGCTTCACCACCTTCTTG3′.

### 2.7. Statistical Analysis

Statistical differences among groups were analyzed using one-way ANOVA and the post hoc Tukey's test. If two groups were analyzed, the significance was determined by Student's *t*-test. Statistical significance was established at 0.05 (^∗^*P* ≤ 0.05, ^∗∗^*P* ≤ 0.01, and ^∗∗∗^*P* ≤ 0.001) using SPSS® software V.21 (Chicago, IL). GraphPad Prism 7.0 was used to prepare the statistical illustrations (GraphPad Software, La Jolla, CA, USA).

## 3. Results

### 3.1. Confirming Aging Markers in Replicative Senescence in Mouse Embryonic Fibroblasts

**Figure 1:**
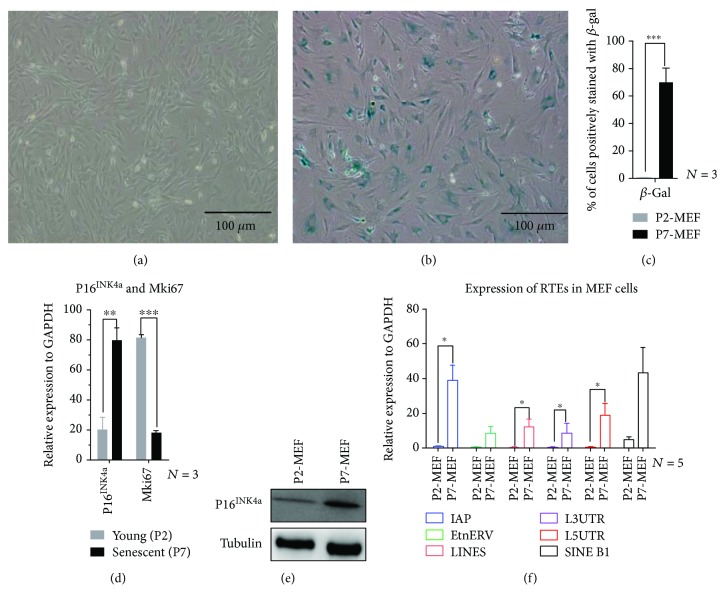
Replicative senescence of MEFs. (a, b) SA-*β*-gal staining of MEFs, comparing young (P2-MEF, in (a)) and senescent (P7-MEF, in (b)). (c) Quantification of *β*-gal-positive (blue stained) MEFs. (d) mRNA expression level of Mki67 and P16^INK4a^ examined by RT-qPCR. (e) P16^INK4a^ protein level in young and senescent MEFs. *β*-Tubulin was used as the loading control. (f) RT-qPCR analysis of different classes of RTEs. ^∗^*P* ≤ 0.05, ^∗∗^*P* ≤ 0.01, and ^∗∗∗^*P* ≤ 0.001.

Before testing the three aging markers *in vivo*, we examined them in replicative senescence. Mouse embryonic fibroblasts (MEFs) were isolated and grown to senescence *in vitro*. We found that P7-MEFs (MEFs at the 7^th^ passage) exhibited an enlarged phenotype with significantly increased *β*-gal staining (Figures 1(a)–1(c)). Furthermore, RT-qPCR showed that Mki67, a marker for cell proliferation, was significantly decreased, while P16^INK4a^ mRNA and protein levels were significantly increased in senescent MEFs (Figures 1(d) and 1(e)). In addition, RNA-Seq was performed on these cells, and we identified a number of lncRNAs and mRNAs that were differentially expressed in senescent MEFs compared to young MEFs (data not shown). We further examined the expression levels of different classes of RTEs (IAP, LINEs, 3 ′UTR of LINEs (L3UTR), and 5′UTR of LINEs (L5UTR)) and observed they were significantly upregulated in senescent MEFs (Figure 1(f)). These observations suggested that replicative senescence in MEFs was associated with increased expression of P16^INK4a^, RTEs, and *β*-gal and could serve as positive controls for these three senescence markers we proposed to use in the *in vivo* tissues.

### 3.2. Aging Brain Revealed Marked Regional Specific Expression of Aging Markers

**Figure 2:**
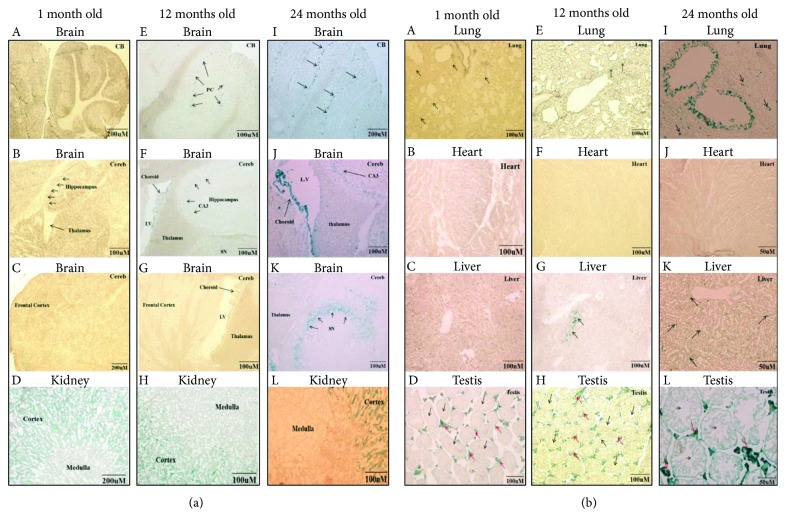
Chronological expression of *β*-gal in the brain, kidney, lung, liver, heart, and testis. (a) The brain and kidney. A–C: sagittal brain sections at 1 month did not show any positive staining signal. E–G: 12 months showed a few positively stained cells within the cerebellum (CB), where PC indicates Purkinje cells (E), and faintly stained cerebrum (Cereb), CA3 of the hippocampus, and choroid plexus (Choroid) (F). I–K: 24-month-old brain sections showed positive *β*-gal staining in the cerebellar folia, specifically in Purkinje cells (PC) (I), choroid plexus of the lateral ventricle (LV) (J), and CA3 (J) and substantia nigra (SN) (K). D, H, L: Photomicrographs of kidney cross-sections at different ages (1 month of age in D, 12 months of age in H, and 24 months of age in L) stained with *β*-gal showed strong signal in the renal cortex at 1 month, 12 months, and 24 months of age, while renal medulla remained unstained. (b) Photomicrographs of lung, liver, heart, and testis sections at different ages (1 month of age in A–D, 12 months of age in E–H, and 24 months of age in I–L) stained with *β*-gal. A, E, and I showed a few scattered positively stained lung cells that were observed in 1-month-old and 12-month-old lungs (arrows), and a strong signal was detected in old mouse lung bronchi (24 months of age), with a few scattered positive cells located in the alveolar epithelium (arrows). B, E, and J showed that no *β*-gal-positive staining was observed at any age examined in the heart. C, G, and K showed a few *β*-gal-positive stained cells in the liver at 12 months and 24 months of age (arrows). D, H, L: Interstitial cells of the testis were strongly positively stained for *β*-gal (red arrows).

**Figure 3:**
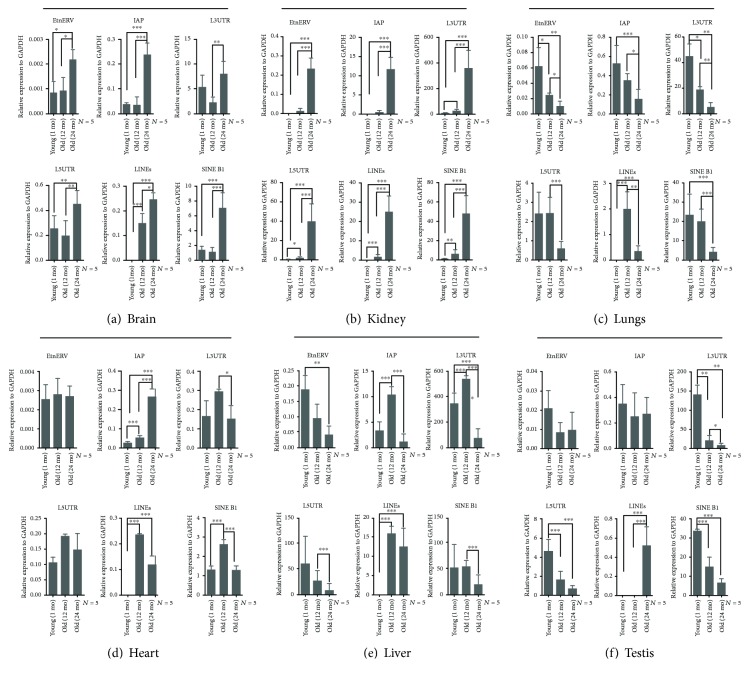
Chronological expression of RTEs (IAP, ERV, L3UTR, L5UTR, LINEs, and SINE B1) in the brain, kidney, lung, liver, heart, and testis. (a) RT-qPCR analysis indicated that all RTEs were significantly upregulated in the aged mouse brain. (b) RT-qPCR analysis of RTEs showing that they were significantly upregulated in aged mouse kidneys. (c) RT-qPCR analysis showed that the RTE expression was significantly downregulated in aged mouse lungs. (d) RT-qPCR analysis of RTEs in the heart showed that only IAP was upregulated, while L3UTR, LINEs, and SINE B1 were downregulated. (e) RT-qPCR analysis of liver RTEs showed that the majority of them were significantly decreased with age. (f) RT-qPCR analysis of RTEs in the testis showed that the majority of them were significantly decreased in aged testes (24 months of age). ^∗^*P* ≤ 0.05, ^∗∗^*P* ≤ 0.01, and ^∗∗∗^*P* ≤ 0.001.

**Figure 4:**
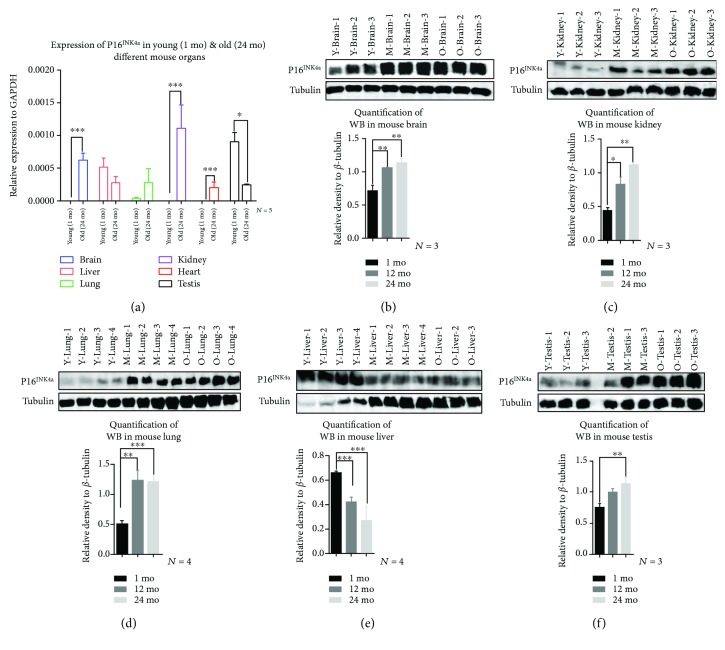
Chronological expression of P16^INK4a^ in different organs. (a) RT-qPCR, of P16^INK4a^ mRNA, in different mouse organs. Significant upregulation was observed in the mouse brain, kidney, and heart. Significant downregulation was observed in testes. No significant changes were observed in the liver or lungs (b–f): P16^INK4a^ protein expression at 1 month (Y), 12 months (M), and 24 months (O) (b) in the brain showed upregulation at M and O compared to Y, (c) in the kidney showed upregulation at M and O compared to Y, (d) in the lungs showed upregulation at M and O compared to Y, (e) in the liver showed downregulation in M and O compared to Y, and (f) in the testis showed upregulation in O compared to Y. The histograms below the panels showed the densitometric mean ± SD normalized to the corresponding level of the loading control protein, *β*-tubulin (^∗^*P* ≤ 0.05, ^∗∗^*P* ≤ 0.01, and ^∗∗∗^*P* ≤ 0.001).

Efforts have been carried out to reveal molecular changes in brain regions that are susceptible to age-associated diseases, including the hippocampus and substantia nigra. For example, Purkinje and cortical hippocampus neurons were positively stained with *β*-gal in aged mice [26]. However, other brain regions are less studied. In the current study, we found that Purkinje cells (Figure 2(a) A, E, I) and hippocampus-specific CA3 region neurons (Figure 2(a) B, F, J) were strongly stained in aged mouse brains compared to young mouse brains. *β*-Gal staining increased with age in the choroid plexus of the lateral ventricles (LV) (Figure 2(a) G, K), substantia nigra (SN) (Figure 2(a) J), and a few scattered cells in the frontal cortex. For RTEs, we found that LINEs were upregulated, which was similar to other studies [27, 28], and all different classes of RTEs were significantly upregulated in the aged mouse brain (Figure 3(a)). In addition, both P16^INK4a^ mRNA (Figure 4(a)) and protein (Figure 4(b)) levels were upregulated in the aged mouse brain. These findings suggested that age-associated brain senescent cells were P16^INK4a^ positive and exhibited increased RTE activity.

### 3.3. Aging Markers Occurred in the Kidney as Early as 1 Month of Age

Aged kidneys are associated with decreased glomerular filtration rate, which is due to the reductions in glomerular capillary plasma flow rate and glomerular capillary ultrafiltration coefficient, and decreased renal blood flow, resulting from the reduction in afferent arteriolar resistance [29]. Mortuza et al. reported that diabetic kidneys showed increased *β*-gal staining and decreased *Sirt1* mRNA levels [30]. We found that the *β*-gal-positive cells were predominantly restricted to the renal cortex, in line with a previous report showing that the renal cortex was more sensitive than the renal medulla [31], and showed high expression of *β*-gal as early as 1 month of age (Figure 2(a) D). The staining intensity increased further with age at 12 months and 24 months (Figure 2(a) H, L), but the renal medulla did not show any staining even at 24 months of age (Figure 2(a) L). As seen in the mouse brain, the kidney showed upregulation of both RTEs (Figure 3(b)) and P16^INK4a^ mRNA (Figure 4(a)) and protein (Figure 4(c)) levels with increasing age. These data indicated that the kidney might be susceptible to aging earlier than the brain and the renal cortex was much more sensitive to aging than the renal medulla.

### 3.4. Changes in Aging Lungs Were Restricted to Bronchioles

There are many age-associated changes in the human respiratory system including shrinkage of the lung volume, reduced pulmonary reserve, and increased susceptibility to pulmonary infectious diseases [32]. Cellular senescence is known to be involved in the pathogenesis of some lung diseases, including idiopathic pulmonary fibrosis (IPF) and chronic pulmonary disease. Fibroblast cells isolated from IPF patients showed upregulation of senescence marker P16^INK4a^, P21, and *β*-gal [33, 34]. We stained lung tissue sections for *β*-gal at 1 month, 12 months, and 24 months. We observed positively stained lung sections in 24-month-old mice, mainly around bronchioles and a few scattered positive cells in the lung alveolar epithelium (Figure 2(b) I), whereas middle-aged mice at 12 months showed only a small amount of low-intensity signal around the bronchioles (Figure 2(b) E). Young mouse lung tissue sections showed very little positive *β*-gal staining (Figure 2(b) A). We quantified mRNA expression levels for several different RTEs and observed significantly decreased expression with age except for LINEs, which increased at 12 months of age, followed by a significant decrease at 24 months of age (Figure 3(c)). Similar to previous reports [31], P16^INK4a^ mRNA did not increase significantly with age (Figure 4(a)) in the lung. Even though others failed to detect the P16^INK4a^ protein level either by IHC or by western blotting [31], our results showed that the P16^INK4a^ protein was significantly upregulated with age (Figure 4(d)). These observations suggested that aging lung tissue was associated with upregulation of P16^INK4a^, positive *β*-gal-stained bronchioles, and decreased expression of RTEs.

### 3.5. Aging Heart Did Not Stain with *β*-Gal but Had Increased P16^INK4a^

Induced cardiac hypertrophy in rats showed increased *β*-gal stain, accumulation of lipofuscin, and high levels of cyclin-dependent kinase inhibitors [35]. Our heart sections stained for *β*-gal however did not reveal any positively stained cells in any of the ages tested (Figure 2(b) B, F, J). Additionally, among the tested RTEs, only IAP was significantly upregulated with aging, while LINEs and SINE B1 were increased at 12 months of age followed by significant downregulation (Figure 3(d)). On the other hand, P16^INK4a^ mRNA was significantly increased in the aged heart (Figure 4(a)). For technical reasons, we could not examine the protein level of the P16^INK4a^. These findings indicated that the mouse heart showed different patterns of aging markers in the aging process.

### 3.6. Aging Liver Was Accompanied by Decreased Accumulation of P16^INK4a^-Positive Cells and Decreased RTEs

In spite of the regenerative properties of liver hepatocytes, aging enhances the vulnerability of the liver to various diseases and advanced age is associated with a poorer prognosis in general. Hepatic steatosis has been associated with increased expression of the senescence marker P21 and telomere-associated DNA damage foci [36]. We found that at 1 month, mouse livers did not show any positive *β*-gal staining (Figure 2(b) C), while 12-month-old mouse (Figure 2(b) G) and 24-month-old mouse (Figure 2(b) K) livers showed a few focally distributed *β*-gal-positive cells. In addition, liver RTEs showed increased expression of IAP and LINEs at the age of 12 months followed by a significant decrease at 24 months of age (Figure 3(e)). Consistent with the observed RTE expression, both P16^INK4a^ mRNA and protein were significantly downregulated in aged liver at 24 months (Figures 4(a) and 4(e)). These observations suggested that the liver might effectively eliminate P16^INK4a^ senescent cells and thus showed no aging phenotype until very old age.

### 3.7. Aging Testes Showed Increased *β*-Gal Staining and 16^INK4a^ Expression

Testicular aging is associated with decreased testosterone levels, which is projected to be 1% every year after 30 years of age in humans [37]. Similarly, the SAMP8 mouse showed age-related decrease in serum testosterone level to 71% at the age of 4 and 12 months of age, compared to a 26% decrease in R1 mice of the same age [38]. Our results showed strong positive *β*-gal staining in testicular interstitial cells, which was increased with age (Figure 2(b) D, H, L). We did not observe any positive *β*-gal staining in the seminiferous tubules (Figure 2(b) D, H, L). Meanwhile, RTEs were significantly decreased with aging with the exception of LINEs, which were significantly increased (Figure 3(f)). In line with what Krishnamurthy et al. has reported [31], P16^INK4a^ mRNA was significantly decreased with aging (Figure 4(a)). However, p16^INK4a^ protein levels were significantly upregulated (Figure 4(f)).

## 4. Discussion

Data accumulated in the last 30 years have helped us to identify different environmental and genetic factors, as well as chemical substances that affect lifespan in many different eukaryotic species [32, 33]. However, understanding the molecular mechanisms of the aging process remains an unsolved question in biology.

Despite multiple theories proposed to explain the aging process, it is still not clear how aging begins and what influence aging exerts on different organs. Therefore, we assessed the response of different mouse organs (brain, liver, lung, kidney, heart, and testis) to three possible mechanisms involved in aging. We examined the expression of *β*-gal staining, P16^INK4a^, and RTEs at three different stages in the aging process (1 month, 12 months, and 24 months). As organisms age, gradual accumulation of unrepaired cellular damage can drive the aging process and determine the incidence of age-related disease [39, 40].

Moreover, jumping of TEs into functional (coding or regulatory) DNA regions can produce loss of function and increase genome instability, resulting in the death of affected cells. The occurrence of transposition events also can lead to various degenerative processes [41]. In spite of the tight regulation of TEs, either epigenetically or posttranscriptionally, the mobility of LINE-1 has been previously reported in the nervous system [19, 42]. Age-related gross morphometric measurements of mouse organs revealed age-associated increase in body weight and percentage of body fat and decreased testicular weights [43]. However, more detailed responses of various body organs to various aging markers have not been carried out before.

Aged mouse brain showed upregulation of the three tested aging markers used in our study. Increased *β*-gal staining in cerebellar Purkinje neurons might reflect locomotor incoordination that is often associated with aged individuals. Increased *β*-gal staining also was observed in the hippocampus and substantia nigra, which are major brain regions associated with neurodegenerative diseases such as Alzheimer and Parkinson diseases, respectively. In addition, human aging is associated with reduced amounts of cerebrospinal fluid (CSF) and increased protein concentrations [23], which might be attributed to an aged choroid plexus. Thus, specific brain regions appear to be highly sensitive to an aging phenotype, which suggested that further investigations are warranted, especially for the choroid plexus and for the unique functions of CSF in healthy people and patients.

Whole genome bisulfide sequencing in the aged human brain revealed that DNA has significant lower methylation compared with that of newborn DNA [24]. In line with previous studies showing that upregulation of IAP-RTEs with aging is due to promoter hypomethylation [25], we observed generalized and significant increases in tested RTEs with and without LTR. In addition, a significant accumulation of p16^INK4a^ mRNA and protein was observed. Together, these observations revealed that the brain is highly sensitive to physiological aging at the molecular level, influencing both morphology and function.

Similar to the brain, mouse kidneys demonstrated significant upregulation of the aging markers used in this study, especially in the renal cortex. It was surprising that the kidneys expressed a senescent phenotype earlier than any of the other organs included in this study. These findings might reflect the essential role of the kidney in the aging process. Previous studies have not focused on this relationship. The kidney is important in maintaining homeostasis of the body, suggesting that aging of the kidney is more likely to occur earlier than other organs and possibly the age-related decline of other organs might be a consequence of failure of the kidney to effectively eliminate circulating age-inducing molecules. Since elderly humans have less renal functional reserve and are more susceptible to chronic renal diseases [44], actions to preserve renal function might help to delay or alleviate aging-related consequences in the whole body.

Since previous studies did not test the lung and the liver for *β*-gal staining and protein expression levels of P16^INK4a^ [31], we demonstrated that *β*-gal staining of bronchioles was associated with aging in the mouse lungs along with upregulation of P16^INK4a^ protein and significant decrease in RTE expression at 24 months of age. Similarly, we observed a few scattered *β*-gal-positive cells in the mouse liver along with significant downregulation of RTEs. These findings suggested that lungs and livers were less influenced by aging or their aging may be a consequence of aging in the kidney. These observations raised a question concerning the theory of aging through retrotransposition. Generalized age-associated declines are thought to affect all organs, but the increased expression of RTEs was observed only in the brain and kidney. This may be due to the fact that the mobility of RTEs is a functional necessity in the brain and kidney and not a function of the aging process. In contrast to the lung, the liver showed downregulation of the P16^INK4a^ protein. Further investigations are required to determine how the mouse liver might eliminate P16^INK4a^-positive cells.

We observed that the aged mouse heart did not present any *β*-gal-positive cells, while IAP was significantly increased with aging. In addition, LINEs and SINE B1 were significantly increased at 12 months of age, followed by a significant decrease at 24 months of age. In line with the observations in the brain and lungs, the mouse heart showed increased expression of P16^INK4a^ mRNA. A greater understanding of the processes involved in cardiac aging may lead to identification of novel and more specific cardiac aging biomarkers.

Similar to kidneys, the interstitial cells of the testes were positively stained for *β*-gal as early as 1 month of age, and the staining presented significant increases with aging. This might attribute to the early development of both organs from intermediate mesoderm through formation of urogenital ridge. However, cells in the seminiferous tubules did not show any positive staining for *β*-gal. This could be associated with the decline of testosterone levels that is associated with aging in males. Additionally, testicular RTEs and P16^INK4a^ mRNA were significantly decreased with aging.

**Figure 5:**
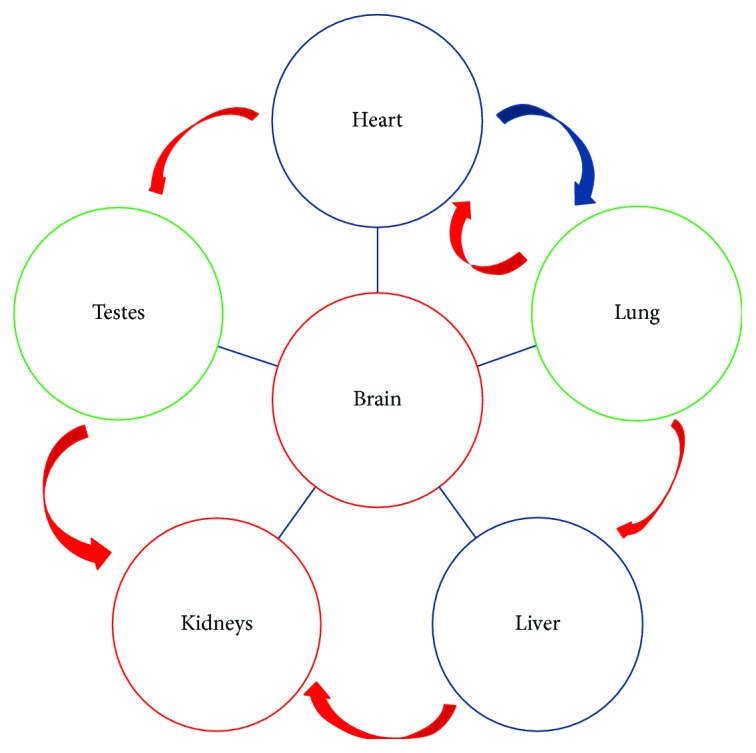
Model of organ aging. The model illustrates the center of aging that might be due to changes in the brain and kidney, then followed by the lung and testis. The heart and liver were the least affected organs. Red arrows represent circulating blood, which is filtered by the kidneys. The blue lines represent neurons that connect organs with the brain.

This work provided the *in vivo* chronological aging profiling in different organs in mice using three aging biomarkers. We demonstrated that aging significantly influenced specific brain regions, the renal cortex, pulmonary bronchioles, and interstitial cells of the testes but had little or no effect on lung parenchyma, the liver, heart, and testicular seminiferous tubules (summarized in Table 1). In conclusion, the gradual functional decline of peripheral organs might be a consequence of the aging brain or kidneys either through aging of neurons that influence these organs or through failure of the kidneys to eliminate age-associated molecules that occur due to environmental and genetic causes (Figure 5). Additionally, the age-dependent changes in RTE expression may be related to changes in function rather than directly associated with the aging process. The upregulation of RTEs in the mouse brain and kidneys might positively enhance the clearance of P16^INK4a^-positive cells. We think that measuring the expression of these aging markers might provide evidence for the process of aging, as upregulation of the three aging markers (P16^INK4a^, *β*-gal, and RTEs) indicated higher aging susceptibility as seen in the brain and kidneys, yet upregulation of P16^INK4a^ and *β*-gal with down- or various regulations of RTEs indicated a moderate aging progress as seen in the lungs and testes, while downregulation of the one or two markers and with less or no *β*-gal staining represent longevity as seen in the liver and heart.

In summary, P16^INK4a^ expression was upregulated with age in all mouse tissues tested except for the liver, thus reinforcing the importance of P16^INK4a^ as a biomarker of *in vivo* aging. However, recent studies demonstrated that P16^INK4a^ is not exclusively expressed in senescent cells [45, 46], which might raise a question about the future use of P16^INK4a^ senolytics and encourage research on more specific aging biomarkers.

The information provided in this study may be useful for consideration of age-associated diseases including neurodegenerative diseases such as Alzheimer disease and Parkinson disease, kidney-associated diseases such as chronic renal failure, pulmonary-associated diseases such as chronic pulmonary diseases, cardiovascular disease, and reproductive diseases associated with the decline of testosterone levels. This study provides a foundation for other aging research groups to use the mouse as an animal model and identify specific organs to elucidate molecular mechanisms underlying the aging process. Taken together, these observations could lead to development of tissue-specific senolytics that enhance senescent cell clearance and/or decrease accumulation in the brain and kidney, which would allow the kidney and brain to remain healthier and sustain function longer with positive effects on later life in people.

The remaining challenge will be to answer the following questions: First, is derepression of RTEs a major driving force for aging or is it merely a consequence of existing pathogenesis? Second, it is important to identify ideal aging biomarkers to be able to better predict the functional capacity and assess the biological process of aging *in vivo*.

## Figures and Tables

**Table 1:** Summary of RTEs in different mouse organs.

Organ	LINES	L3UTR	L5UTR	IAP	SINE B1	EtnERV
Brain	+++	+++	+++	+++	+++	+++
Kidneys	+++	+++	+++	+++	+++	+++
Lungs	+(12 mo) - (24 mo)	---	---	---	---	---
Heart	+(12 mo) - (24 mo)	---	Ns	++	+(12 mo) - (24 mo)	Ns
Liver	+(12 mo) - (24 mo)	+(12 mo) - (24 mo)	---	+(12 mo) - (24 mo)	---	--
Testes	+++	---	---	Ns	---	Ns

(+) indicates upregulation; (-) indicates downregulation; Ns indicates no significant changes were observed.

## Data Availability

The authors confirm that the data supporting the findings of this study are available within the article.
